# Evaluating semantic relations in neural word embeddings with biomedical and general domain knowledge bases

**DOI:** 10.1186/s12911-018-0630-x

**Published:** 2018-07-23

**Authors:** Zhiwei Chen, Zhe He, Xiuwen Liu, Jiang Bian

**Affiliations:** 1 Department of Computer Science, Florida State University, Tallahassee, FL, USA; 2 School of Information, Florida State University, 142 Collegiate Loop, Tallahassee, FL, 32306 USA; 3 Department of Health Outcomes and Biomedical Informatics, University of Florida, Gainesville, FL, USA

**Keywords:** Word embedding, Semantic relation, UMLS, WordNet

## Abstract

**Background:**

In the past few years, neural word embeddings have been widely used in text mining. However, the vector representations of word embeddings mostly act as a black box in downstream applications using them, thereby limiting their interpretability. Even though word embeddings are able to capture semantic regularities in free text documents, it is not clear how different kinds of semantic relations are represented by word embeddings and how semantically-related terms can be retrieved from word embeddings.

**Methods:**

To improve the transparency of word embeddings and the interpretability of the applications using them, in this study, we propose a novel approach for evaluating the semantic relations in word embeddings using external knowledge bases: Wikipedia, WordNet and Unified Medical Language System (UMLS). We trained multiple word embeddings using health-related articles in Wikipedia and then evaluated their performance in the analogy and semantic relation term retrieval tasks. We also assessed if the evaluation results depend on the domain of the textual corpora by comparing the embeddings of health-related Wikipedia articles with those of general Wikipedia articles.

**Results:**

Regarding the retrieval of semantic relations, we were able to retrieve semanti. Meanwhile, the two popular word embedding approaches, Word2vec and GloVe, obtained comparable results on both the analogy retrieval task and the semantic relation retrieval task, while dependency-based word embeddings had much worse performance in both tasks. We also found that the word embeddings trained with health-related Wikipedia articles obtained better performance in the health-related relation retrieval tasks than those trained with general Wikipedia articles.

**Conclusion:**

It is evident from this study that word embeddings can group terms with diverse semantic relations together. The domain of the training corpus does have impact on the semantic relations represented by word embeddings. We thus recommend using domain-specific corpus to train word embeddings for domain-specific text mining tasks.

## Background

Mining useful but hidden knowledge from unstructured data is a fundamental goal of text mining. Towards this goal, the field of natural language processing (NLP) has been rapidly advancing, especially in the area of word representations. In the past few years, neural-network-based distributional representations of words such as word embeddings have been shown effective in capturing fine-grained semantic relations and syntactic regularities in large text corpora [1–3]. Therefore, they have been widely used in deep learning models such as those for text classification and topic modeling[4, 5]. For example, convolutional neural networks (CNNs) have become increasingly popular for modeling sentences and documents [6]. Recurrent neural networks (RNNs), especially bidirectional long short-term memory (LSTM) models, have been used to train language models for tasks such as machine translation and information extraction [7]. Nevertheless, the vector representations of word embeddings, with poor interpretability, still act as a black box in downstream applications [8]. Improving the interpretability of word embeddings has been a challenge due to the high dimensionality of these models. Recent research is mostly focused on constructing neural networks but not on the interpretations of word embeddings [1, 3, 9, 10].

**Fig. 1:**
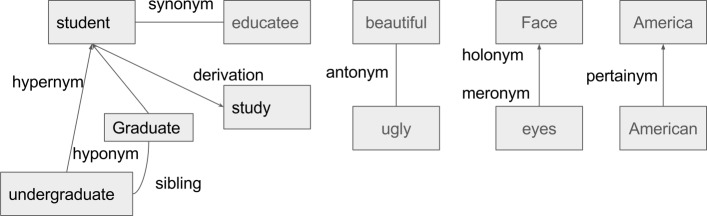
Examples of semantic relations

Semantic relations are the meaningful associations between words or phrases [11]. In a widely-used open domain lexicon, WordNet, there are nine semantic relations. Table 1 shows the definitions of these semantic relations. Figure 1 gives concrete examples of these semantic relations. In the medical domain, medical relations are prevalent between medical terms. For example, nausea *has a symptom* flu; breast cancer *has a finding site* breast. With the advancement of health IT, many medical concepts and their relations have been encoded in biomedical ontologies and controlled vocabularies, which have been used for electronic health records, semantic reasoning, information extraction, and clinical decision support [12].

**Table 1:** Semantic relation definition in WordNet

Relation	Definition
Synonym	A term with exactly or nearly the same meaning as another term. For example, *heart attack* is a synonym of *myocardial infarction*
Antonym	A term with an opposite meaning with another term, e.g., *big : small*, *long : short*, and *precede : follow*
Hypernym	A term with a broad meaning under which more specific words fall. For example, *bird* is a hypernym of *pigeon*, *crow*, *eagle*, and *seagull*
Hyponym	A term of more specific meaning than a general or superordinate term applicable to it. It is the opposite of hypernym. For example, *pigeon*, *crow*, *eagle* and *seagull* are hyponyms of *bird*
Holonym	A term that denotes a whole whose part is denoted by another term„ e.g., *body* is a holonym of *arm*
Meronym	A term that denotes part of something but which is used to refer to the whole. It is the opposite of holonym
Sibling	The relationship denoting that terms have the same hypernym. E.g., *son* and *daughter* are sibling terms, since they have the same hypernym *child*
Derivationally related forms	Terms in different syntactic categories that have the same root form and are semantically related, e.g., *childhood* is a derivationally related form of *child*
Pertainym	Adjectives that are usually defined by phrases such as “of or pertaining to” and do not have antonyms, e.g., *America* is a pertainym of *American*

Identifying the semantic relations between words and phrases is the basis for understanding the meaning of the text [13]. Therefore, investigating semantic relations represented in word embeddings has the potential to improve the transparency of word embeddings and the interpretability of the downstream applications using them. Nevertheless, semantic relations in word embeddings have not been adequately studied, especially in the biomedicine domain. In this study, we explore the semantic relations (i.e., semantically related terms) in the neural word embeddings using external knowledge bases from Wikipedia, WordNet [13], and Unified Medical Language System (UMLS) [14]. We formulated two research questions (RQ) as:

- RQ1: How are the different types of semantic relations distributed in the word embeddings?
- RQ2: Does the domain of the training corpus affect the distribution of the domain-specific relations in the word embeddings?

To investigate how different semantic relations are represented in word embeddings, we first need to obtain the semantic relations from the real world. To construct gold-standard semantic relation datasets, we used two lexical knowledge bases: WordNet and Unified Medical Language System (UMLS). WordNet is a large lexical database of English. It groups nouns, verbs, adjectives, and adverbs into sets of cognitive synsets, each expressing distinct concepts [13]. The UMLS is a compendium of over 200 controlled vocabularies and ontologies in biomedicine [14]. It maps over 10 million terms into over three million medical concepts such that the terms with the same meaning are assigned the same concept ID. The terms of the same concept are considered as synonyms with each other.

We trained word embeddings with three popular neural word embedding tools (i.e., Word2Vec [2], dependency-based word embeddings [10], and GloVe [3]) using over 300,000 health-related articles in Wikipedia. To answer RQ1, we evaluated these embeddings in two semantic relation retrieval tasks: 1) the analogy term retrieval task and 2) the relation term retrieval task. To answer RQ2, we sampled the same number of general articles from Wikipedia, used them to train the word embeddings using Word2vec, and evaluated the embeddings in two semantic relation retrieval tasks with the same evaluation dataset.

In our recent conference paper [15], we explored this problem with only health-related Wikipedia articles as the training corpus and evaluated different word embeddings with 10 general semantic relations in the semantic relation retrieval tasks. In this extended paper, we significantly expanded our analysis in the following aspects. First, besides training word embeddings with the health-related Wikipedia articles, we also trained the embeddings with general Wikipedia articles to assess the impact of the domain of the corpus on the evaluation results. Second, we expanded our gold-standard evaluation dataset with 6 medical relations from the UMLS. With this, we can better understand how medical relations are represented in the word embeddings. Third, we added the analogy term retrieval task for both the general semantic relations as well as medical relations. This allowed us to compare our results with previous published results which also evaluated word embeddings using analogy questions. Fourth, we visualized how different semantic relation terms are distributed in the word embedding space with respect to a particular term. This will help us better understand the intrinsic characteristics of the word embeddings. This study has the potential to inform the research community on the benefits and limitations of using word embeddings in their deep-learning-based text mining and NLP projects.

## Related work

### Neural word embeddings

In an early work, Lund et al. [16] introduced HAL (Hyperspace Analogue to Language), which uses a sliding window to capture the co-occurrence information. By moving the ramped window through the corpus, a co-occurrence matrix is formed. The value of each cell of the matrix is the number of co-occurrences of the corresponding word pairs in the text corpus. HAL is a robust unsupervised word embedding method that can represent certain kinds of semantic relations. However, it suffers from the sparseness of the matrix.

In 2003, Bengio et al. [17] proposed a neural probabilistic language model to learn the distributed representations for words. In their model, a word is represented as a distributed feature vector. The joint probability function of a word sequence is a smooth function. As such, a small change in the vectors of the sequence of word vectors will induce a small change in probability. This implies that similar words would have similar feature vectors. For example, the sentence *“A dog is walking in the bedroom”* can be changed to *“A cat is walking in the bedroom”* by replacing dog with cat. This model outperforms the N-gram model in many text mining tasks with a big margin but suffers from high computational complexity.

Later, Mikolov et al. [18] proposed the Recurrent Neural Network Language Model (RNNLM). RNNLM is a powerful embedding model because the recurrent networks incorporate the entire input history using the short-term memory recursively. It outperforms the traditional N-gram model. Nevertheless, one of the shortcomings of RNNLM is its computational complexity in the hidden layer of the network. In 2013, Mikolov et al. [1, 2] proposed a simplified RNNLM using multiple simple networks in Word2vec. It assumes that training a simple network with much more data can achieve similar performance as more complex networks such as RNN. Word2vec can efficiently cluster similar words together and predict regularity relations, such as *“man is to woman as king is to queen”*. The Word2vec method based on skip-gram with negative sampling [1, 2] is widely used mainly because of its accompanying software package, which enabled efficient training of dense word representations and a straightforward integration into downstream models. Word2vec uses techniques such as *negative sampling* and *sub-sampling* to reduce the computational complexity. To a certain extent, Work2vec successfully promoted word embeddings to be the *de facto* input in many recent text mining and NLP projects.

Pennington et al. [3] argued that Word2vec does not sufficiently utilize the global statistics of word co-occurrences. They proposed a new embedding model called GloVe by incorporating the statistics of the entire corpus explicitly using all the word-word co-occurrence counts. The computational complexity is reduced by including only non-zero count entries. In [3], GloVe significantly overperforms the Word2vec in the semantic analogy tasks.

Most of the embedding models used the words surrounding the target word as the context based on the assumption that *words with a similar context have the similar meanings* [19]. Levy and Goldberg [10] proposed a dependency-based word embeddings with the argument that syntactic dependencies are more inclusive and focused. They assumed one can use syntactic dependency information to skip the words that are close but not related to the target word, meanwhile capturing the distantly related words that are out of the context window. Their results showed that dependency-based word embeddings captured less topical similarity but more functional similarity.

To improve the interpretability of Word2vec, Levy and Goldberg [20] illustrated that Word2vec implicitly factorizes a word-context matrix, whose cells are the pointwise mutual information (PMI) of the respective word and context pairs, shifted by a global constant. Arora et al. [21] proposed a generative random walk model to provide theoretical justifications for nonlinear models like PMI, Word2vec, and GloVe, as well as some hyper-parameter choices.

### Evaluation of word embeddings

Lund and Burgess’s experiments based on HAL [16] demonstrated that the nearest neighbors of a word have certain relations to the word. However, they did not investigate the specific types of relations that these nearest neighbors have with the word. Mikolov et al. [1] demonstrated that neural word embeddings could effectively capture analogy relations. They also released a widely used analogy and syntactic evaluation dataset. Finkelstein et al. [22] released another widely used dataset for word relation evaluation, *WordSim-353*, which provides obscure relations between words rather than specific relations.

Ono et al. [23] leveraged supervised synonym and antonym information from the thesauri as well as the objectives in Skip-Gram Negative Sampling (SGNS) model to detect antonyms from unlabeled text. They reached the state-of-the-art accuracy on the GRE antonym questions task.

Schnabel et al. [24] presented a comprehensive evaluation method for word embedding models, which used both the widely-used evaluation datasets from Baroni et al. [2, 25] and a dataset manually labeled by themselves. They categorized the evaluation tasks into three classes: *absolute intrinsic*, *coherence*, and *extrinsic*. Their method involves extensive manual labeling of the correlation of words, for which they leveraged crowdsourcing on the Amazon Mechanical Turk (MTurk). In our study, we investigated the relations among terms in an automated fashion.

Levy and Goldberg [20] showed that the skip-gram with negative sampling can implicitly factorize a word-context matrix, whose cells are the Pointwise Mutual Information (PMI) of the corresponding word and its context, shifted by a constant number, i.e., *M*_*PMI*_− log(*k*), where *k* is the negative sampling number. Later, they [26] systematically evaluated and compared four word embedding methods: PPMI (Positive Pointwise Mutual Information) matrix, SVD (Singular Value Decomposition) factorization PPMI matrix, Skip-Gram Negative Sampling (SGNS), and GloVe, with nine hyperparameters. The results showed that none of these methods can alway outperform the others with the same hyperparameters. They also found that tuning the hyperparameters had a higher impact on the performance than the algorithm chosen.

Zhu et al. [27] recently examined Word2vec’s ability in deriving semantic relatedness and similarity between biomedical terms from large publication data. They preprocessed and grouped over 18 million PubMed abstracts and over 750k full-text articles from PubMed Central into subsets by recency, size, and section. Word2vec models are trained on these subtests. Cosine similarities between biomedical terms obtained from the word2vec models are compared against the reference standards. They found that increasing the size of dataset does not always enhance the performance. It can result in the identification of more relations of biomedical terms, but it does not guarantee better precision.

### Visualization of word embeddings

Recently, Liu et al. [28] presented an embedding technique for visualizing semantic and syntactic analogies and performed tests to determine whether the resulting visualizations capture the salient structure of the word embeddings generated with Word2vec and GloVe. Principal Component Analysis projection, Cosine distance histogram, and semantic axis were used as the visualization techniques. In our work, we also explored other types of relations that are related to medicine, e.g., morphology, finding site. Google released Embedding Projector [29] which includes PCA [30] and t-SNE [31] as an embedding visualization tool in the TensorFlow framework [32].

## Methods

To explore the semantic relations in word embeddings, we used three tools to generate the embeddings, namely, Word2vec [2], dependency-based word embeddings [10], and GloVe [3]. Meanwhile, we obtained two training corpora from Wikipedia: one was a health related corpus, the other was the corpus of a random sample of the entire Wikipedia. We then used WordNet [13] and the UMLS [14] to evaluate performance of these word embeddings through 1) the analogy term retrieval task and 2) the relation term retrieval task.

To better explain our methods, we first list the key terminologies used in this section:

- Lemma: A lemma is the canonical form, dictionary form, or citation form of a set of words. For example, *sing*, *sings*, *sang* and *singing* are forms of the same lexeme, with *sing* as the lemma.
- Relation term: A term (word or phrase) that is associated to another term with a semantic relation. For example, *beautiful* is an antonym of *ugly*. They are relation terms of each other.
- Evaluation term: An evaluation term is a word or phrase that is used to retrieve its relation terms in its nearest neighbors in the vector space of the word embeddings.

### Dataset collection and preprocessing

#### Training datasets

We first obtained the entire Wikipedia English dataset collected on 01/01/2017. To obtain the health-related corpus, we fetched all the subcategories of *Health* within the depth of 4 in the category hierarchy of Wikipedia, using the web-tool *PetScan* [33]. Then we filtered out the articles that were not categorized into any of the subcategories. To obtain a comparable general topic corpus, we randomly sampled the same number of articles from the entire Wikipedia corpus.

#### Analogy evaluation datasets

We first constructed a general analogy evaluation dataset with over 9000 analogy questions using the analogy term gold standard from [1, 2, 9]. In addition, we constructed medical-related analogy evaluation dataset with over 33,000 medical-related analogy questions using six relations in the UMLS.

#### UMLS relations

In this study, we used five popular and well-maintained source vocabularies, including SNOMED CT, NCIt (National Cancer Institute Thesaurus), RxNorm, ICD (International Classification of Diseases), and LOINC (Logical Observation Identifiers Names and Codes). The UMLS also contains medical relations between concepts. In this study, we studied six medical relations in UMLS, i.e., *may treat*, *has procedure site*, *has ingredient*, *has finding site*, *has causative agent* and *has associated morphology*. We fetched these relations from the 2015 AA release of the UMLS. As we did not use phrases in this study, we filtered out the relations with more than one word in the concept names. We also removed all the punctuations and converted the concept names to lowercase.

#### WordNet relations

For the semantic relations in WordNet, we employed Natural Language Toolkit (NLTK) [34] to extract semantic relations for the evaluation terms. The details of specific procedures we used are listed separately [35]. For each evaluation term, we first obtained the synset of the term and then searched for semantic relations based on the synset.

### Word embedding training configuration

For the word embedding methods we investigated in this study, Word2vec, dependency-based word embeddings are based on the same neural network architecture *Skip-gram model*. To reduce the bias, we used the same Skip-gram model configuration when constructing these word embeddings. We chose the commonly used parameters in previous publications [1, 3, 9, 10]. The dimension of the vector was set to 300. The size of the context window was set to 5 (i.e., five words before and after the target word). The negative sample was 5 and the subsampling threshold was 1e-4. The number of iterations was 15 and the number of threads was 20. For dependency-based word embeddings, we used the tool from the authors’ website [36]. For the Word2vec, we used the tool from its project page [37].

For GloVe, although it employs the same idea of using the target word to predict its context, it uses a different training method called *adaptive subgradient* [38]. We therefore used the same configuration as the experiment in [3]. The dimension of the vector was set to 300. The window size was set to 5, the *x*_*max*_ to 100, and the maximum iteration to 50. We also used 20 threads to train the model with the tool downloaded from on GloVe’s official website [39].

### Evaluation methods

We evaluated the performance of different word embeddings in retrieving relation terms for the evaluation terms. Our evaluation method consists of two tasks, corresponding to four evaluation datasets.

1. *Analogy term retrieval task*, with evaluation gold-standard datasets:
  General analogy questions previously used in evaluating word embeddings [1, 2, 9], which include six subtasks: *capital-common*, *capital-world*, *currency*, *city-in-state*. *family*, and *airlines*.
  Medical related analogy questions, i.e., pairs of terms with the same UMLS relations. For example, the term pairs (*atropine*, *uveitis*) and (*rifampin*, *tuberculosisare*) have the same UMLS relation *may treat*. We constructed an analogy question (*atropine*, *uveitis* :: *rifampin*, ?), for which the answer is *tuberculosisare*.
2. *Relation term retrieval task*, with evaluation datasets:
  General semantic relations: 10 subtasks, 9 from the 9 semantic relations in WordNet and one from the synonyms in the UMLS.
  Medical relations, which include the aforementioned six medical relations in the UMLS.

#### Analogy term retrieval tasks

One of the most appealing characteristics of Word2vec is that it is capable of predicting analogy relations. A well-known example is “man is to woman as king is to queen.” Given the embedding vectors of three words: *king*, *queen* and *man*, we can use a simple algebra equation, $\overrightarrow {king} - \overrightarrow {queen} = \overrightarrow {man} - \overrightarrow {woman}$, to predict the fourth word *woman*. The relation between these two pairs of words is the analogy relation. We consider the analogy relation as a special type of semantic relations. It is different from the other semantic relations (e.g., synonym, hypertym, hyponym) in WordNet and UMLS defined in the Background section. To evaluate the analogy relation in different word embeddings, we collected general analogy questions from [1, 2, 9] (syntactical relations were not included). As shown in Table 2, the analogy evaluation dataset includes a total of 9331 questions in six different groups of analogy relations. We also constructed medical related analogy questions as shown in Table 3. All the analogy questions are in the same format: given the first three words, predict the fourth word using vector arithmetic and the algorithm *nearest neighbor*. In other words, given the words *a*, *b*, *c* and *d*, 1) use the vectors of the first three words in the embedding, $\vec {a}$, $\vec {b}$ and $\vec {c}$; to compute the predicted vector $\vec {d'}$ using the formula

1 $$ \vec{d'} = \vec{c} - \vec{a} + \vec{b},   $$

**Table 2:** The information about the general analogy dataset

Question group	Count	Percentage
Capital-common	506	5.24%
Capital-world	4524	48.48%
Currency	866	9.28%
cCty-in-state	2467	26.44%
Family	506	5.42%
Airlines	462	4.95%
Total	9331	100.00%

**Table 3:** The information about the medical related evaluation dataset

Subtask	# of relations^1^	# of analogy	# of relation
		questions	questions
may-treat	595	10,000	595
has-procedure-site	43	903	43
has-ingredient	57	1596	57
has-finding-site	369	10,000	369
has-causative-agent	47	1081	47
has-associated-morphology	184	10,000	184
Total	1295	33,585	1,295

^1^1: # indicates *the number of*

2) identify the nearest *k* neighbors of $\vec {d'}$ in the embedding vector space using cosine similarity, namely *set*(*d*_1_,*d*_2_,…,*d*_*k*_). If word *d* is in *set*(*d*_1_,*d*_2_,…,*d*_*k*_), the result of a question was considered as a true positive case, otherwise it is a false positive case. We computed the accuracy of each question in each group as well as the overall accuracy across all the groups.

Note that most of the previous evaluation of word embeddings using the analogy questions focused on the top 1 nearest neighbor [1, 3, 9, 10, 26]. In this study, we expanded the analysis to top 1, 5, 20 and 100 nearest neighbors. This reason is that some words have multiple synonyms. As such, the correct answer in the gold standard may not be retrieved as the top 1 nearest neighbor. Another reason is that the training dataset is a small part of the whole corpus in Wikipedia and the training process of word embeddings involves many rounds of random sampling. Therefore, the resulting embeddings are not deterministic.

#### Relation term retrieval tasks

The relation term retrieval tasks consisted of general relation term retrieval tasks and medical relation term retrieval tasks.

1. General Relation Term Retrieval Tasks

The general semantic relation term retrieval task included ten subtasks, nine of which each corresponding to the nine semantic relations in WordNet [13] and one of which corresponding to the synonyms in the UMLS [14] (i.e., the terms with the same Concept Unique Identifiers (CUI) in the UMLS). While the relations from WordNet represent the general semantic relations, we also used the UMLS synonyms to investigate the performance of retrieving synonyms in the medical domain from word embeddings.

Our semantic relation evaluation dataset focused on nouns and adjectives, which was based on the statistics of synsets in WordNet [40], where 69.79% (82,115/117,659) of the synsets were nouns, and 15.43% (18,156/117,659) of synsets were adjectives. We constructed the evaluation dataset in four steps: 1) We employed a named entity recognition tool developed in our lab, *simiTerm* [41], to generate all the candidate evaluation terms based on the N-gram model. 2) We filtered out the noisy terms including:

- Terms with more than four words;
- Terms with a frequency < 100 in the corpus;
- Terms starting or ending with a stop word (We used the default stop word list in *simiTerm*);
- Unigrams that are not noun, adjective, or gerund;
- Multi-grams not ending with a noun or a gerund.

We obtained 38,560 candidate evaluation terms after the second step. 3) We matched the candidate evaluation terms with terms in the UMLS and WordNet and kept those that can be mapped to least one term in the UMLS or at least one synset in WordNet. After the third step, we retained 22,271 terms as the *evaluation terms*. 4) For every evaluation term, we identified its *relation terms*, i.e., *UMLS synonyms*, *synonyms*, *antonyms*, *hypernyms*, *hyponyms*, *holonyms*, *meronyms*, *siblings*, *derivationally related forms* and *pertainyms* in WordNet.

At the end, we obtained a gold standard dataset for the semantic relations with 22,271 evaluation terms with at least one out of ten relations. Table 4 shows the basic characteristics of these evaluation terms. The column *# of Evaluation Terms* shows that the numbers of terms for different relations are unbalanced. *Hypernyms* and *synonyms* are the most frequent relation terms, whereas *pertainyms* and *antonyms* are the least frequent relation terms. Column *Average* is the total number of relation terms divided by the number of evaluation terms. We give examples of the evaluation terms and their relation terms in Table 5.

**Table 4:** Information about general semantic relation evaluation dataset

Subtasks	# of evaluation	Percentage^1^	Relation	Average^2^
	terms		terms #	
UMLS synonym	9,235	41.47%	9,211	3.14
Synonym	15,591	70.01%	48,030	4.25
Antonym	2,225	9.99%	2,977	1.38
Hypernym	16,400	73.64%	58,154	4.85
Hoponym	9,168	41.17%	112,215	19.84
Holonym	4,694	21.08%	9,944	3.69
Meronym	3,191	14.33%	13,056	7.14
Sibling	13,993	62.83%	869,814	86.38
Derivation	9,620	43.20%	27,782	2.92
Pertainym	926	4.16%	987	1.20
Total	22,271	38.19%^*^	1,168,921	9.35

^1^Percentage of evaluation terms that has at least one term with the relation

^2^Average number of relation terms that this type of evaluation terms has

^*^Average of all the subtasks

**Table 5:** Examples in the semantic relation evaluation dataset

Evaluation term	UMLS Synonym	WordNet Synonym	WordNet Antonym	WordNet Hypernym	WordNet Hyponym
native american (AN ^*^)	first_nation	amerindian		someone	carib
		indian		individual	arawak
		amerind		mortal	american_indian
hand (N)	hand_no	hired_hand		manual_laborer	right
		deal		help	hooks
		paw		crewman	ostler
important (A)		authoritative	unimportant		
		crucial	noncrucial		
		significant	insignificant		
Evaluation term	WordNet Holonym	WordNet Meronym	WordNet Sibling	WordNet Derivation	WordNet Pertainym
native american (AN)			gatekeeper	amerind	american_indian
			scratcher		
			bereaved		
hand (N)	timepiece	arteria_digitalis	day_labourer	handwrite	
	timekeeper	metacarpus	botany	paw	
	human_being	thenar	printing_process	scriptural	
important (A)				importance	
				cruciality	
				significance	

^*^A: Adjective; N: Noun; AN: Adjective + Noun

2. Medical Relation Term Retrieval Tasks

The medical relations were extracted from the relations table in the UMLS (MRREL). We chose six medical relations in the medical domain including, *may treat*, *has procedure site*, *has causative agent*, *has finding site*, *has associated morphology* and *has ingredient*. The relations are represented as triplets, *(source, relation, target)*, where *source* and *target* are the terms associated with the *relation*. For example, in the relation triplet *(atropine, may treat, uveitis)*, *atropine* is a medication, while *uveitis* is a disease. The relation *may treat* represents that we may use *atropine* to treat *uveitis*. Table 6 gives examples for these six medical relations.

**Table 6:** Examples in the medical relation evaluation dataset

Source	Target	Source	Target	Source	Target
Has associated morphology		Has causative agent		May treat	
Enteritis	Inflammation	Coinfection	Organism	Atropine	Uveitis
Keratosis	Lesion	Coccidiosis	Protozoa	Rifampin	Tuberculosis
Asthma	Obstruction	Asbestosis	Asbestos	Naproxen	Inflammation
Has procedure site		Has ingredient		Has finding site	
Splenectomy	Spleen	Dressing	Foam	Rickets	Cartilage
Keratoplasty	Cornea	Beeswax	Waxes	Pyometra	Uterus
Bronchoscopy	Bronchi	Cellulose	Regenerated	Overbite	Cartilage

To evaluate the performance of word embeddings, for each evaluation term *t* in the dataset, first, we obtained its vector $\vec {t}$ in the embedding, found the top *k* nearest neighbors of $\vec {t}$ in the embeddings using cosine similarity, and fetched the *k* corresponding terms to construct *set*(*t*_1_,*t*_2_,…,*t*_*k*_). Second, we computed the number of relation terms of the evaluation term *t* in *set*(*t*_1_,*t*_2_,…,*t*_*k*_). If the number is greater or equals to one, the evaluation term is a true positive case for the corresponding semantic relation, otherwise it is a false positive case. Third, we computed the *retrieved ratio* of each semantic relation as well as the average *retrieved ratio* for all semantic relations. The key measure of the semantic relation evaluation performance is *retrieved ratio (RR)*, which is defined as

2 $$\begin{array}{*{20}l} hit(e, rel) &= \left\{\begin{array}{ll} 1, \text{ if} (N_{e} \cap e.rel) \neq \emptyset, \\ 0, \text{otherwise}, \end{array}\right.  \end{array} $$

3 $$\begin{array}{*{20}l} RR(rel) &= \frac{\sum_{e \in E} hit(e,rel)}{|\{e: e \in E \land e.rel \neq \emptyset\}|},  \end{array} $$

where *E* is the set of evaluation terms. *e* is an evaluation term in *E*. *N*_*e*_ is the set of nearest neighbors for *e* in the word embedding. *rel* is a semantic relation. *e.rel* is the set of relation terms in *e* w.r.t. *rel*. *hit*(*e,rel*) computes the number of evaluation terms with at least one relation term in its nearest neighbors. The denominator of *retrieved*-*ratio* is the number of evaluation terms with at least one relation term w.r.t. *rel*. The *retrieved ratio* indicates the probability of a relation term occurring in the nearest neighbors of an evaluation term.

Since the number of relation terms for different semantic relations varies, Eq. 3 may be unfair for the semantic relations with much fewer relation terms than others. Therefore, we also use a weighted function to adjust the retrieved ratio:

4 $$\begin{array}{*{20}l} weight(e, rel, n) &= \frac{n}{min(k, |e.rel|)},  \end{array} $$

5 $$\begin{array}{*{20}l} WRR(rel) &= \frac{{\sum\nolimits}_{e \in E} weight(e,rel, hit(e,rel))}{|\{e: e \in E \land e.rel \neq \emptyset\}|},  \end{array} $$

where *k* is the number of nearest neighbors and |*e.rel*| is the number of relation terms in *e* w.r.t. *rel*. *weight*(*e,rel,n*) is used to balance the effect of large number of relation terms.

## Results

In this section, we describe the evaluation results and our analyses. We first give the basic statistics of the corpus and the statistics about the dependency relations. Note that here We only show the statistics of health related Wikipedia corpus. The general Wikipedia corpus exhibits the similar characteristics. For RQ1, using the health related corpus, we investigated the performance of three word embeddings, i.e., Word2vec, dependency-based word embeddings, and GloVe, on both the analogy term retrieval task and the relation term retrieval task. For RQ2, we compared the performance of Word2vec between health related Wikipedia corpus and the general Wikipedia corpus.

### Basic information of the health related Wikipedia corpus

#### General statistics

Our health-related text corpus contained 322,339 English health related articles in Wikipedia including 36,511 subcategories of health, which constitutes about six percent of the entire English Wikipedia. It contained 282,323,236 words. On average, each article contained 876 words.

#### Dependency relation information

For the experiment of dependency-based word embeddings, we used the same corpus from Wikipedia. We obtained 466,096,785 word-context pairs as the training data. Table 7 shows the frequency of the relations. The relation *nmod (noun phrase)* was the most frequent one. Other noun-related relations, such as *compound* and *amod (adjective modifier)* also occurred frequently in the corpus.

**Table 7:** Frequency of the dependency relations in the corpus

Relation name [42]	Frequency	Percentage
nmod	31,740,495	14.44
case	30,937,477	14.07
det	22,721,509	10.34
compound	21,640,690	9.84
amod	16,400,313	7.46
nsubj	14,417,633	6.56
conj	10,616,184	4.83
dobj	10,527,741	4.79
cc	8,437,745	3.84
advmod	7,251,191	3.30

### Visualization embedding space

For the visualization, we tried both PCA and T-SNE methods. We found PCA is more appropriate for our case for the following reasons:

1. In our visualization, we want to show the clusters as well as the linear arithmetic relations between words (e.g., analogy relation). T-SNE is an effective method to show the clusters in the dataset but it does not keep the linear relation between words. PCA satisfies both requirements.
2. PCA is a deterministic method while T-SNE is not. We found that even using the same parameters, the result of T-SNE varied a lot.
3. Most papers we cited in this study used PCA as their visualization method.

**Fig. 2:**
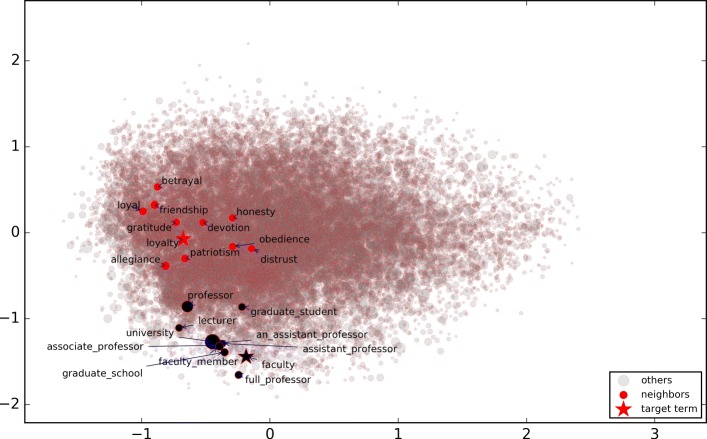
Words *faculty* and *loyalty* and their top 10 nearest neighbors in the reduced 2-D space of the embedding space

**Fig. 3:**
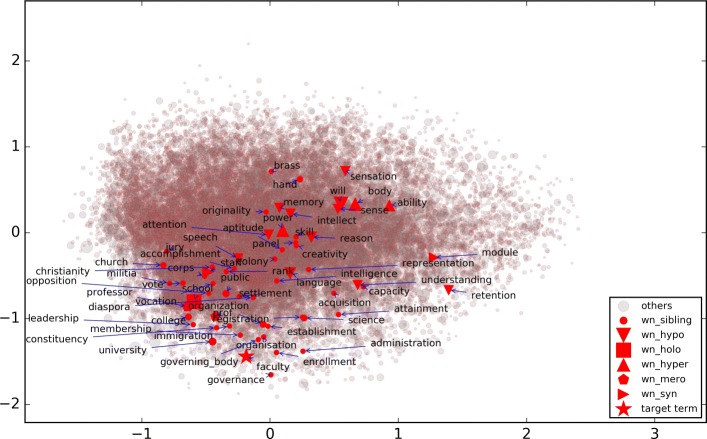
Relation terms of *faculty* in the reduced 2-D space of the embedding space

Figure 2 shows the words *faculty* and *loyalty* and their top 10 nearest neighbors in the reduced word embedding space by Principal Component Analysis (PCA). In Fig. 2, the bigger the circle for the term, the closer it is to the target word in the original (unreduced) embedding space. It shows that although PCA keeps the nearest neighbors relatively close, it does not preserve the distance ranking in the reduced space. In Fig. 3, the same type of relation terms are labeled with the same symbol. The top 10 nearest neighbors of evaluation term *faculty* included a *meronym* “professor” and a *sibling* “university”. Note that 10 nearest neighbors constitute very small sample of the words, considering the size of the vocabulary. It demonstrates that various kinds of relation terms can be retrieved in the nearest neighbors of a given term in the word embedding space.

### Evaluation with the health related Wikipedia corpus

#### Evaluation results of the analogy term retrieval task

**Fig. 4:**
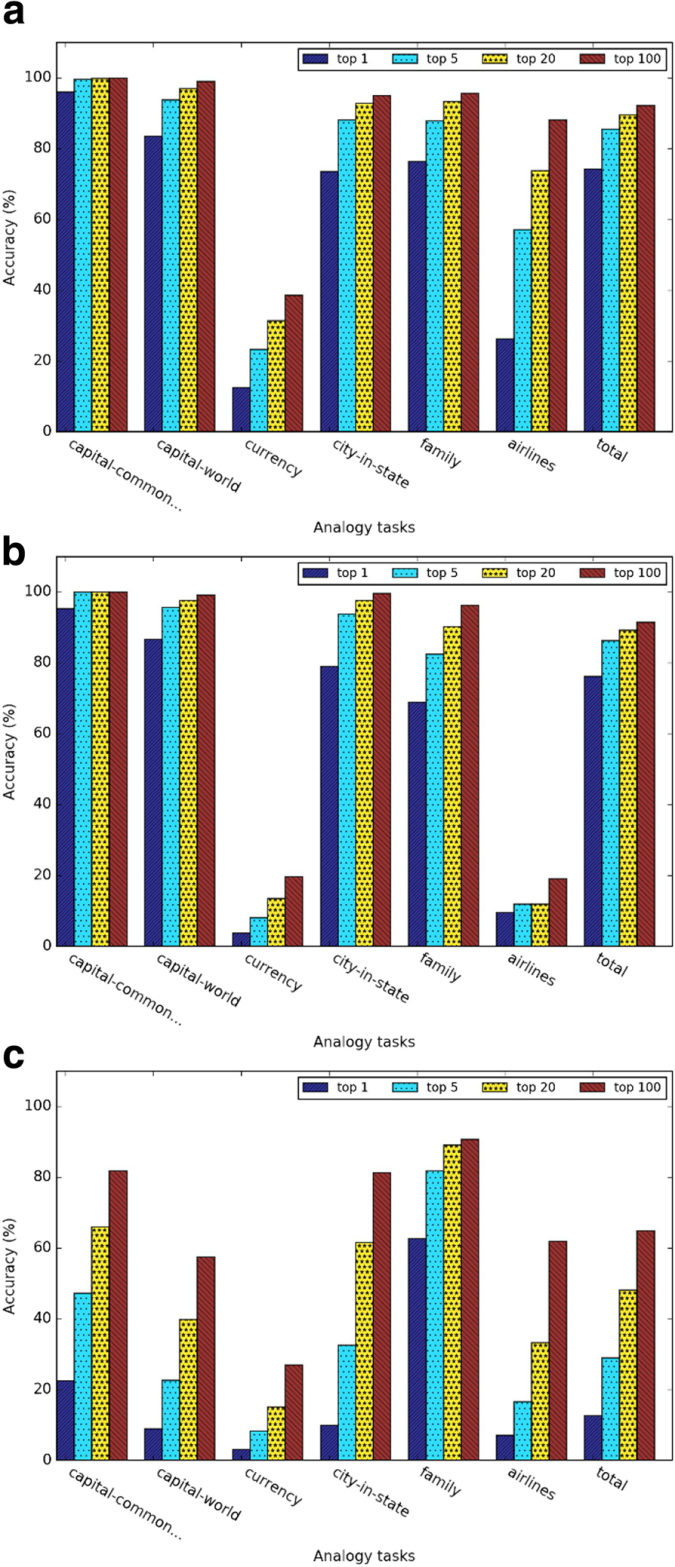
Evaluation results of the general analogy tasks for (**a**) Word2vec, (**b**) GloVe, and (**c**) dependency-based word embeddings

**Fig. 5:**
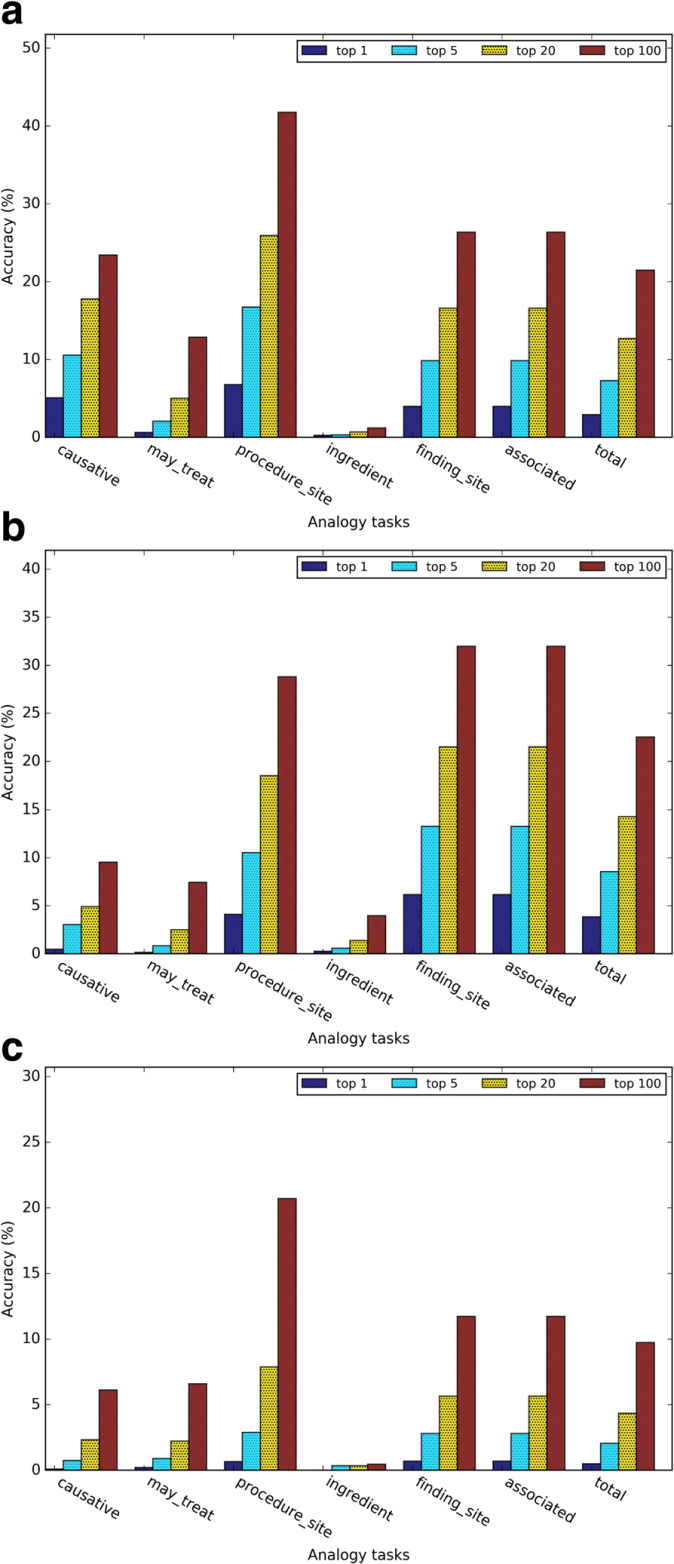
Evaluation results of the medical-related analogy tasks for (**a**) Word2vec, (**b**) GloVe, and (**c**) dependency-based word embeddings

Figures 4 and 5 show the evaluation results of different types of word embeddings in the general and medical-related analogy term retrieval subtasks. We assessed top 1, 5, 20 and 100 nearest neighbors. As the number of retrieved nearest neighbors *k* increases, the accuracy of all the subtasks increases, which is intuitive. However, the performance gain decreases as the number of the nearest neighbors increases. It demonstrates that the closer the term is to the predicted vector, the more likely it is a correct answer.

Figures 5a, b, and c show the evaluation results of the medical-related analogy term retrieval task. In Word2vec and dependency-based word embeddings, the analogy questions on the relation *has procedure site* had the highest accuracy. In GloVe-based embeddings, the analogy questions on relation *has finding site* and *has associated morphology* had a higher accuracy than *has procedure site*. Similar as the general analogy term retrieval task, as the number of retrieved nearest neighbor *k* increases, the accuracy of all the subtasks increases. In addition, the performance gain also increases as the number of the nearest neighbors increases.

**Fig. 6:**
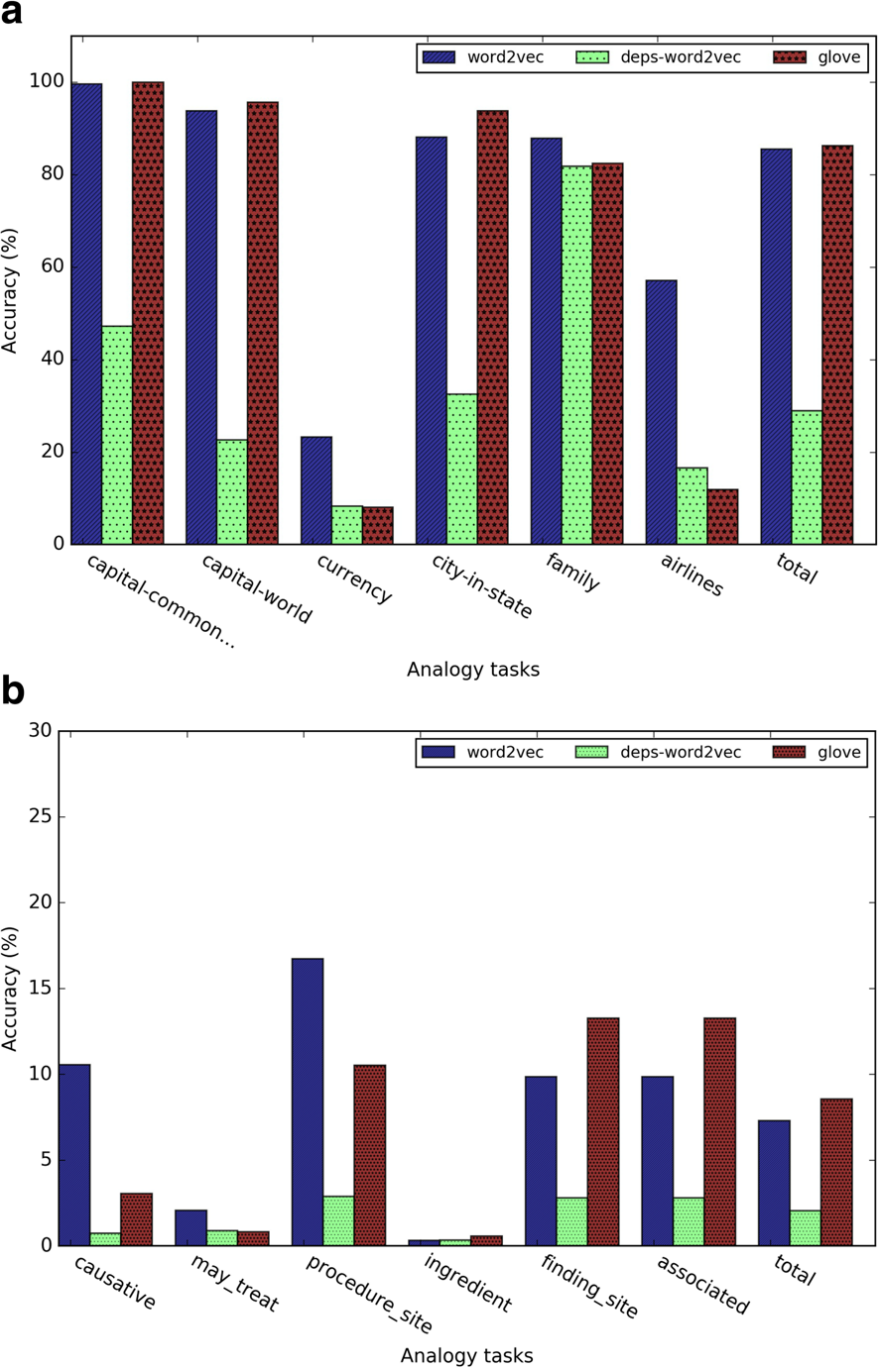
Results of (**a**) the general analogy term retrieval task and (**b**) the medical-related analogy term retrieval task when *k*=5

Figure 6 shows the detailed analogy task results when *k* = 5 for different embedding training methods. In both general analogy term retrieval task and medical-related analogy term retrieval task, the dependency-based word embeddings preformed much worse than other methods. As Levy et al. [10] pointed out, dependency-based word embeddings catch less topic related information than Word2vec. GloVe achieved slightly higher overall accuracy than Word2vec, which weakened the conclusion of [3] that *GloVe outperformed Word2vec 75% on the analogy task*. This discrepancy may be due to the smaller dataset we used in this study. According to Fig. 6a, the performance of the subtasks *currency* and *airlines* are much worse than other subtasks. This may be because health-related articles in Wikipedia may not contain rich information about currency and airlines. Figure 6b demonstrated that word embeddings are able to capture medical-related analogy relations, but the medical-related analogy subtask obtained much worse performance than general analogy subtask.

#### Evaluation results of the semantic relation retrieval task

**Fig. 7:**
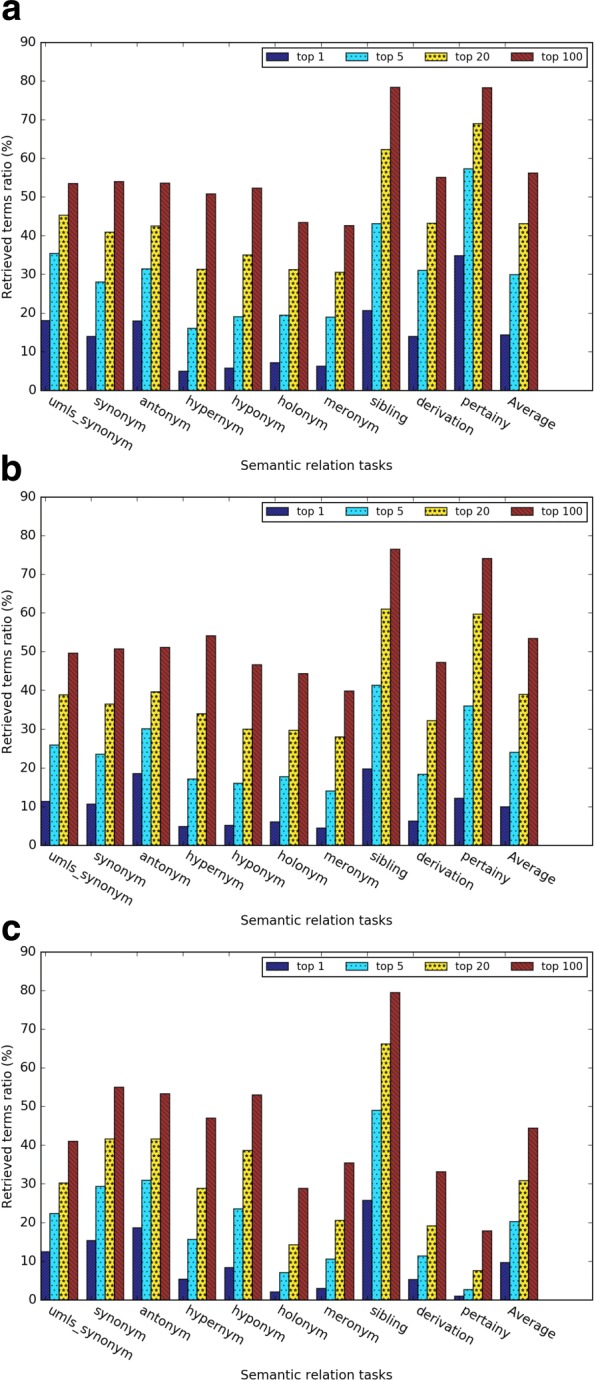
Results of the general relation term retrieval tasks for (**a**) Word2vec, (**b**) GloVe, and (**c**) dependency-based word embeddings

**Fig. 8:**
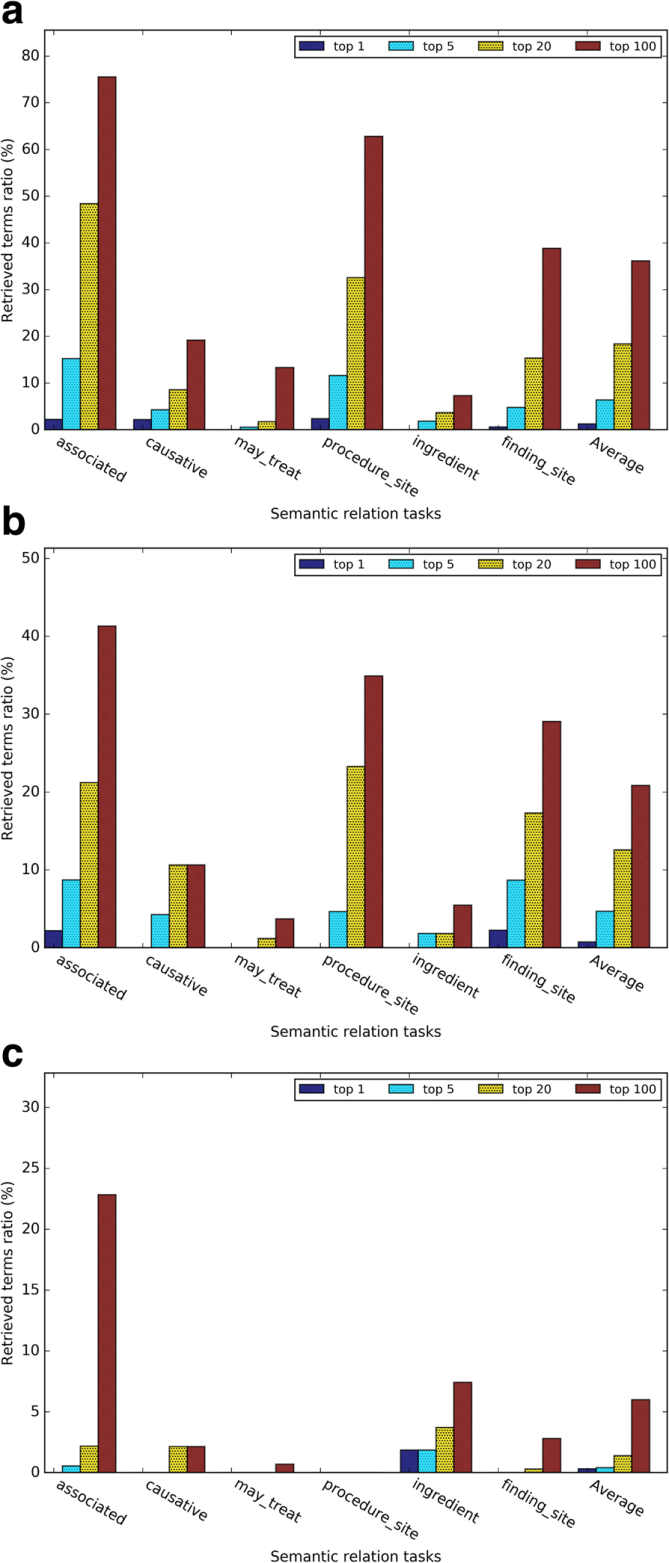
Results of the medical relation term retrieval tasks for (**a**) Word2vec, (**b**) GloVe, and (**c**) dependency-based word embeddings

In this evaluation, we investigated the *retrieved ratio* for top 1, 5, 20 and 100 nearest neighbors for each of the three kinds of word embeddings. As shown in Figs. 7 and 8, as the number of nearest neighbor *k* increases, the retrieved ratio changes much faster than the analogy task. We speculated that the reason is that for each evaluation term, there are more than one relation term, whereas for the analogy task, there is only one correct answer.

Figures 8a, b, and c give the evaluation results for the medical related semantic relation retrieval tasks. Word2vec outperformed GloVe and dependency-based word embeddings. In Word2vec and GloVe, the semantic relations of “has associated morphology” and “has procedure site” had a better retrieved ratio than other semantic relations.

**Fig. 9:**
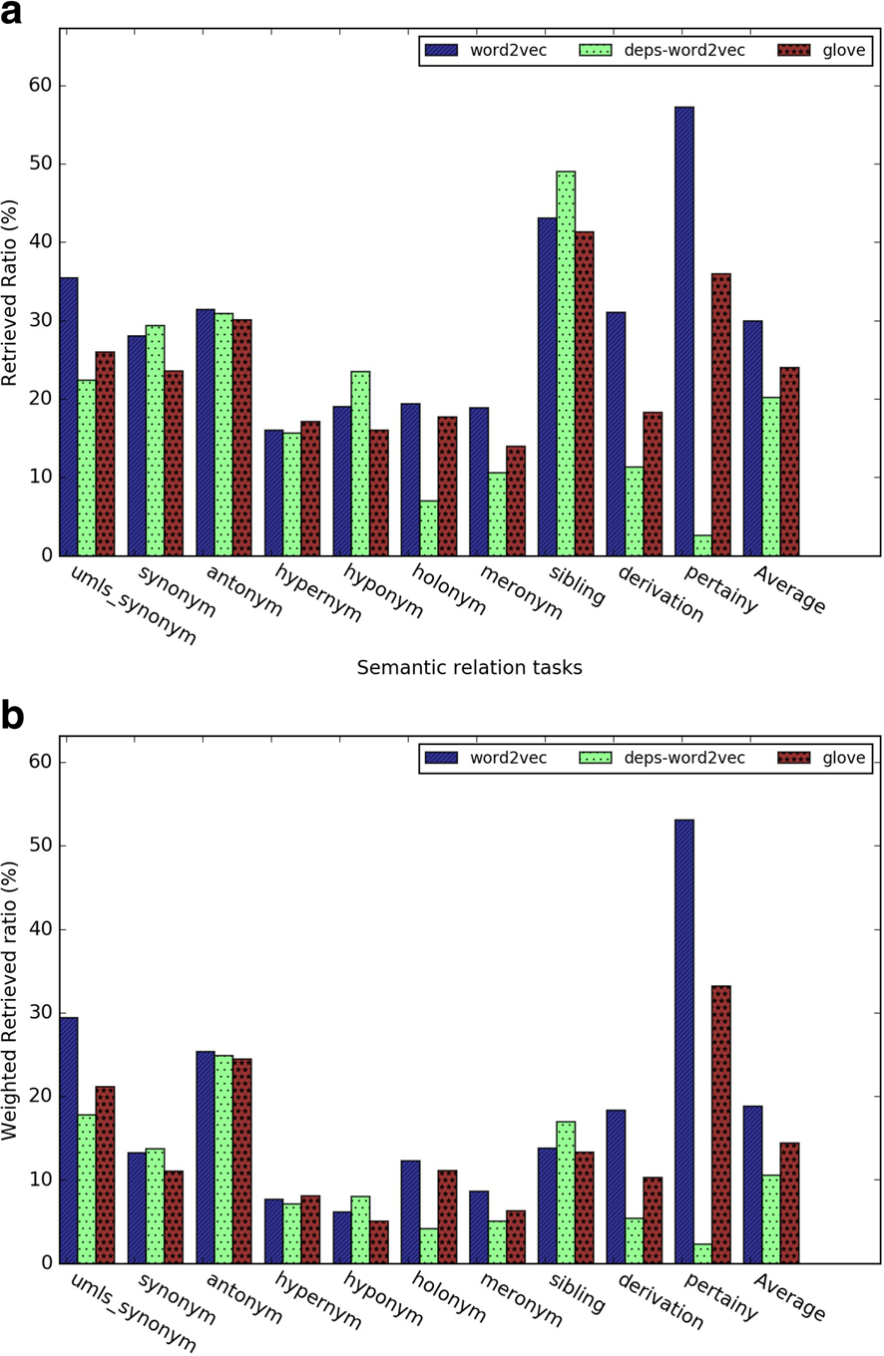
(**a**) The retrieved ratio and (**b**) the weighted retrieved ratio of the relation term retrieval task when *k*=5

**Fig. 10:**
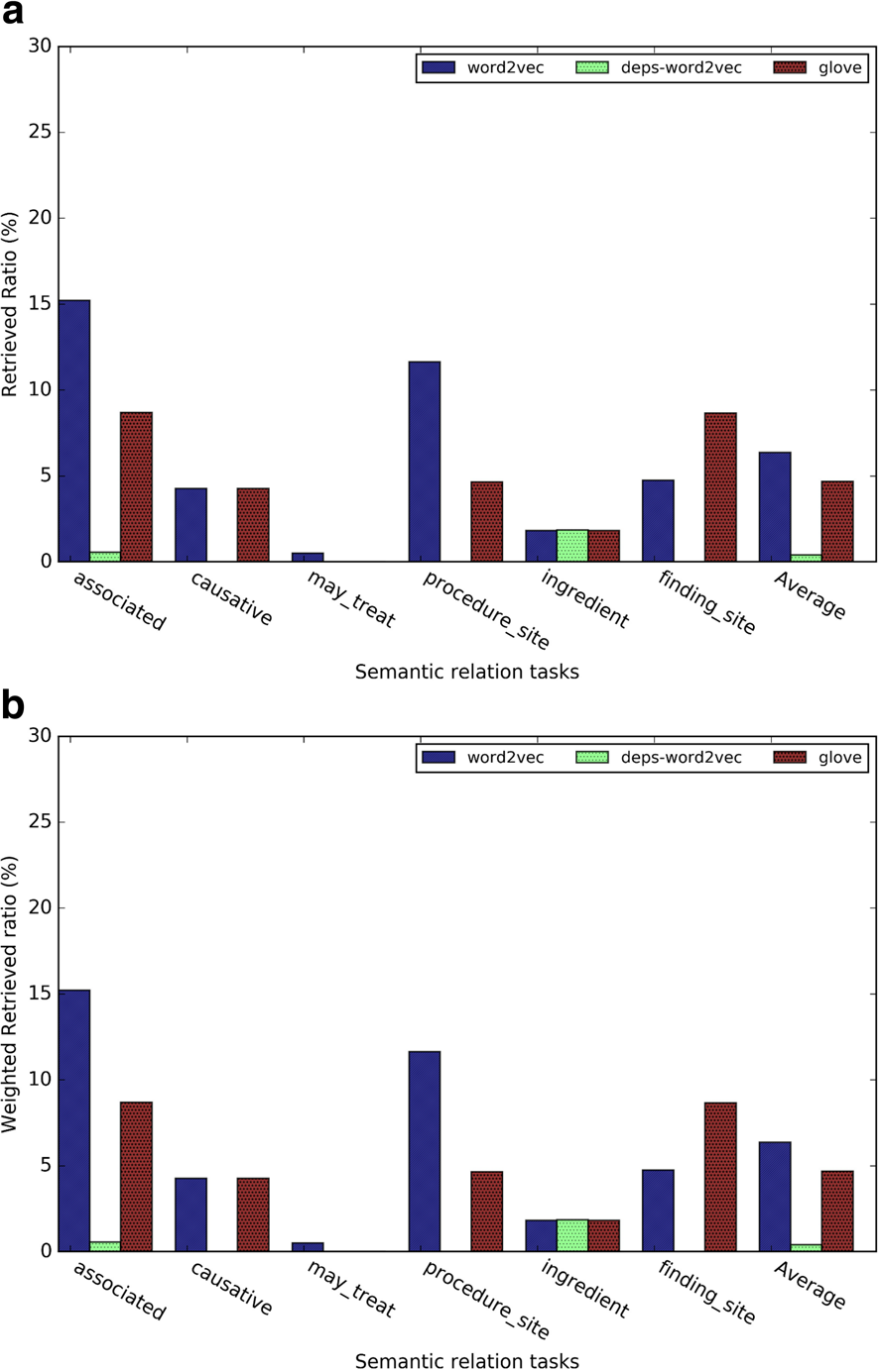
(**a**) The retrieved ratio and (**b**) the weighted retrieved ratio of the medical relation term retrieval task when *k*=5

Figures 9 and 10 give a closer comparison among the methods when *k* = 5. The subtask *sibling relation* achieved the highest retrieved ratio, since there are much more sibling terms than other relations (see Table 4). The *pertainym relation* had the highest retrieved ratio even though there are fewer pertainyms than other types of relation terms. The *weighted retrieved ratio* further (see Fig. 9b) demonstrated that pertainyms have higher probability to be retrieved in the nearest neighbors. Using weighted-retrieved ratio, we found that the WRR of antonyms is even higher than that of the synonyms. Others semantic relations have roughly similar probability to be retrieved in the nearest neighbors. Figure 9a shows dependency-based word embeddings had much worse performance than other methods on the subtasks *pertainym*, *derivation*, *meronym* and *holonym*. Meanwhile, it had a slightly higher performance on subtasks *sibling* and *synonym*. GloVe obtains a slightly worse performance for almost all the subtasks than Word2vec. For medical relation task, we found similar result from Fig. 10a. Word2vec outperformed GloVe in five out of 6 task and dependency based word embedding obtained worst performance. For the medical-related relation term retrieval, the WRR of all the relations are consistently low, which shows that terms with the medical-related semantic relation terms are usually not in the nearest neighbors of a given evaluation term.

### Performance comparison between health related and general Wikipedia corpora

**Fig. 11:**
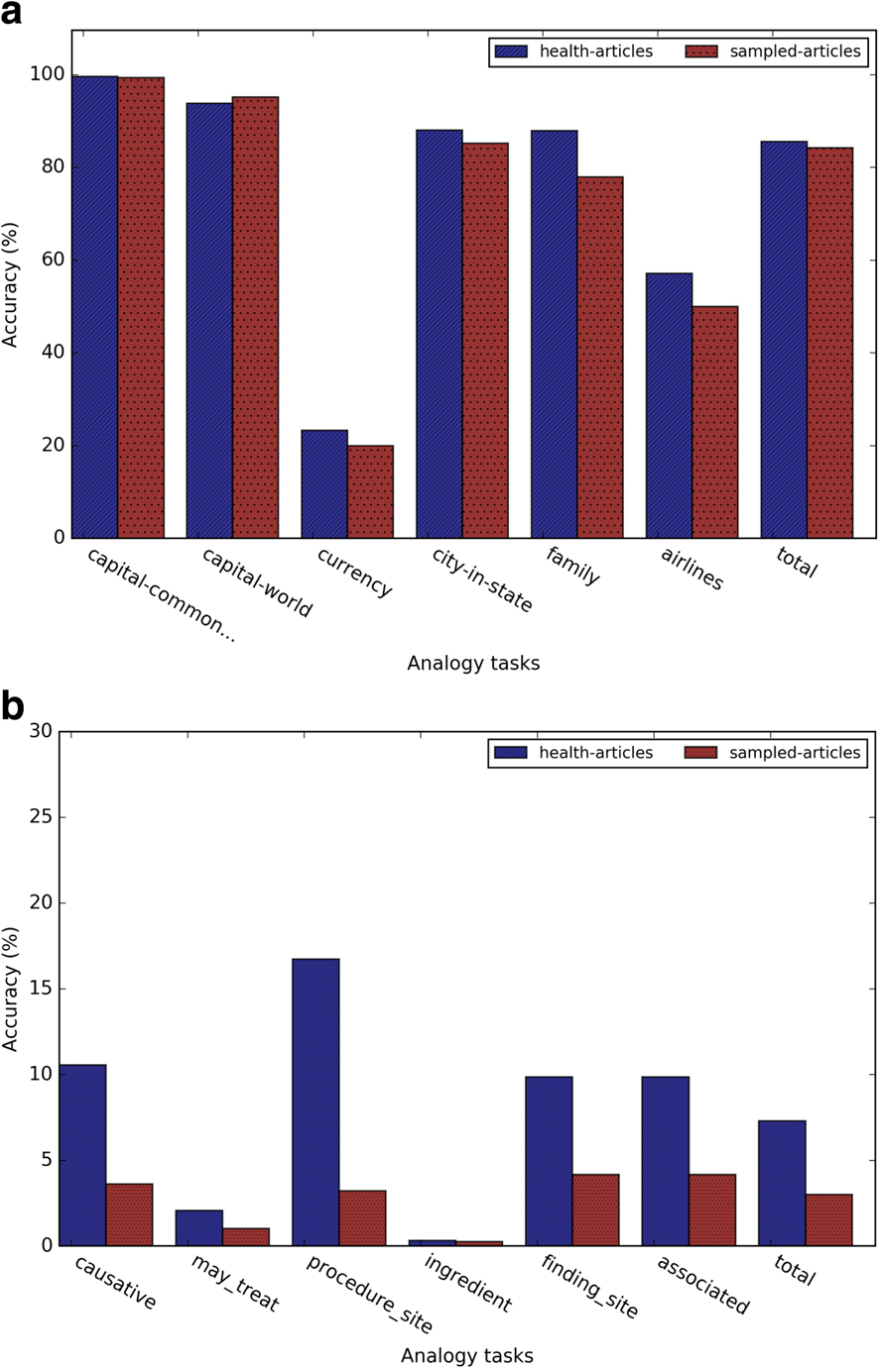
Results of (**a**) the general analogy task and (**b**) the medical-related analogy task using the health-related corpus and the general corpus (*k*=5)

**Fig. 12:**
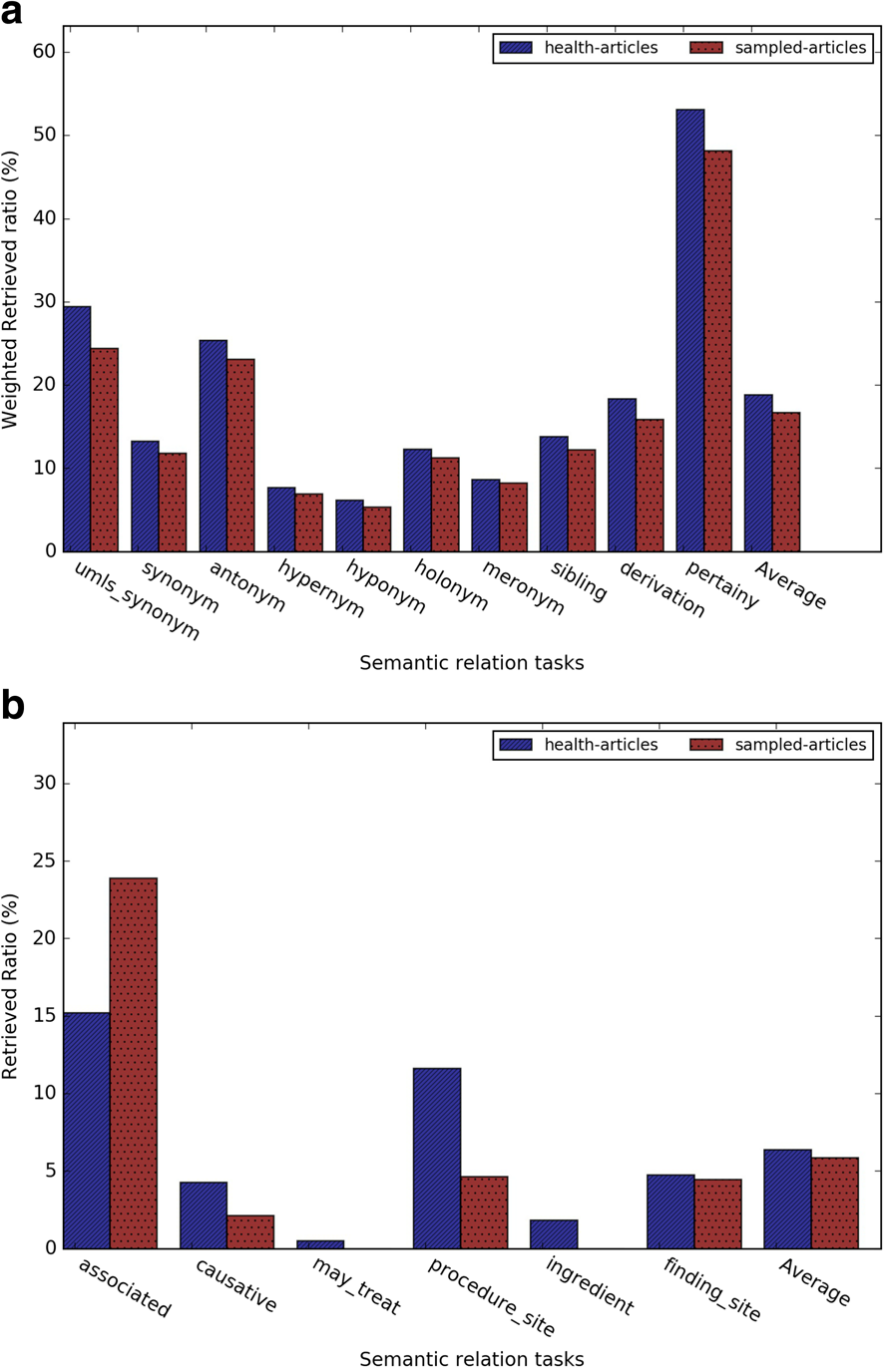
Results of (**a**) the general semantic relation retrieval task and (**b**) the medical-related semantic relation retrieval task using the health-related corpus and the general corpus (*k*=5)

To investigate the impact of the training corpus on our analysis, we also trained Word2vec on general Wikipedia articles, and then compared the analogy and semantic relation tasks between the embeddings trained by two corpora. Figure 11 shows that health related corpus and general corpus had the similar results in the analogy term retrieval task. Figure 11b shows that health related corpus obtained much higher accuracy on the medical analogy questions. Figure 12 shows that health related corpus obtained slightly better result on general semantic relation retrieval task. For the medical related semantic relation evaluation dataset, comparing to result of the general corpus, Fig. 12b shows that the health related corpus obtained much better result on the relations *has causative agent*, *has procedure site*, and *has ingredient*, while obtaining the similar results on the relation *has finding site*, and worse result on the relation *has associated morphology*.

## Discussions

In this study, we used external knowledge bases from Wikipedia, WordNet,and the UMLS to evaluate the semantic relations in different neural word embeddings, namely, Word2vec, GloVe, and dependency-based word embeddings. According to the results, we found that terms with certain semantic relations, such as pertainyms, have a higher likelihood to occur in the nearest neighbors of a given term. With respect to different word embedding tools, we found that GloVe performed slightly better than Word2vec in some analogy term retrieval tasks (e.g., capital-world and city-in-state), but worse in other tasks (e.g., family, currency, and airlines), which is slightly different from [3], possibly due to the smaller training corpora we used. The performance of dependency-based word embeddings in the analogy term retrieval task is worse than Word2vec and GloVe in most of the subtasks except for airline. Even though we used health-related Wikipedia data to train the word embeddings, the comparative results on non-health-related subtask categories should still reflect their relative performance. In the medical-related analogy task, the analogy questions on *has finding site* and *has associated morphology* had a high accuracy in GloVe-based embeddings than Word2vec and dependency-based embeddings. The questions medical-related analogy task had a lower accuracy than the questions in general analogy task. Medical-related analogy questions using dependency-based embeddings had the worst accuracy.

In the relation term retrieval task, we found that the retrieved term ratios of synonyms and antonyms were almost identical. Using weighted-retrieved ratio, we found that the WRR of antonyms was even higher than that of the synonyms. The medical relation term retrieval tasks had a poor WRR, showing that medically-related terms are not in the nearest neighbors for a given evaluation term. The medical relation term retrieval task had a better WRR on *has associated morphology*, *has procedure site*, and *has finding site*. The dependency-based word embeddings had the worse performance among the three. This evaluation also showed that the performance of word embeddings may vary across different domains and different text corpora. Therefore, we suggest that researchers should evaluate different word embeddings using standard evaluation methods such as the ones we conducted in this work before deciding on a particular one to be used.

This study has some limitations. Even though we only employed the cosine similarity as the distance measure to define the nearest neighbors of a word in the embeddings in this study, the nearest neighbors in the embeddings can change (substantially), depending on the definition of distance between word vectors. Euclidean distance and cosine similarity are widely used, while another intuitive distance function is the shortest path, which considers the embedding as a weighted graph and words are connected to each other. The nearest neighbors are the words that can be reached from the evaluation terms with the shortest path. In the future, we will explore the semantic relation distribution in the nearest neighbors defined by other distance methods such as the shortest path. Another interesting direction is to investigate why synonyms and antonyms have similar occurrences in the nearest neighbor of a word. As co-occurred words in the corpora do not necessarily capture semantic relations, it is important to understand the limitations and remedy their impacts on the downstream applications. In this paper, we only used the unigrams in the word embedding training and the gold standard datasets from WordNet and UMLS. In the future, we will investigate how phrase composition may impact the retrieval of semantically related terms in the word embeddings.

## Conclusions

In this study, we evaluated the semantic relations in different neural word embeddings using external knowledge bases from Wikipedia, WordNet,and the UMLS. We trained word embeddings using both health-related and general domain Wikipedia articles. We used the semantic relations in WordNet and UMLS, which covered most of the commonly used semantic relations. We compared the distribution of relation terms in the nearest neighbors of a word in different word embeddings. It is evident that word embeddings can group terms with diverse semantic relations together. The domain of the training corpus has an impact on the semantic relations represented by word embeddings.

## Abbreviations

CNN: Convolutional neural network
CUI: Concept unique identifier
HAL: Hyperspace analogue to language
ICD: International classification of diseases
LSTM: Long short-term memory
LOINC: Logical observation identifiers names and codes
NER: Named entity recognition
NLP: Natural language processing
PPMI: Positive pointwise mutual information
PMI: Pointwise mutual information
POS: Part of speech
RNN: Recurrent neural network
RNNLM: Recurrent neural network language model
RR: Retrieved ratio
SGNS: Skip-Gram negative sampling
SVD: Singular value decomposition
UMLS: Unified medical language system
WRR: Weighted retrieved ratio
