# Correction to: Evaluating semantic relations in neural word embeddings with biomedical and general domain knowledge bases

**DOI:** 10.1186/s12911-018-0655-1

**Published:** 2018-08-22

**Authors:** Zhiwei Chen, Zhe He, Xiuwen Liu, Jiang Bian

**Affiliations:** 1 Department of Computer Science, Florida State University, Tallahassee, FL USA; 2 School of Information, Florida State University, 142 Collegiate Loop, Tallahassee, FL 32306 USA; 3 Department of Health Outcomes and Biomedical Informatics, University of Florida, Gainesville, FL USA

## Correction

After publication of this supplement article [1], it was brought to our attention that the Results section of the abstract contained a partial sentence. The partial sentence is as follows:

“Regarding the retrieval of semantic relations, we were able to retrieve semanti.”

This sentence should be:

“Regarding the retrieval of semantic relations, we were able to retrieve diverse semantic relations in the nearest neighbors of a given word.”
